# The Effects of a Crosslinking Agent on the Microrheological Properties and Cellular Structure of Silicone Rubber Foam Prepared via a Green Process

**DOI:** 10.3390/ma17030707

**Published:** 2024-02-01

**Authors:** Hongyu He, Lulu Li, Hong Liu, Bin Luo, Zhipeng Li, Wenhuai Tian

**Affiliations:** 1 School of Materials Science and Engineering, University of Science and Technology Beijing, Beijing 100083, China; hehongyu666@126.com (H.H.); 15733153822@163.com (L.L.); 2 Guangdong Homeen Organic Silicon Material Co., Ltd., Zhaoqing 526072, China; liuhong@homeen.cn (H.L.); roben@hm-sil.com (B.L.)

**Keywords:** chemical foaming technology, crosslinking agent, in situ examinations, silicone rubber foam, microrheological properties

## Abstract

Chemical foaming technology is widely used in the preparation of silicone rubber foam and is attributable to its one-step molding capability and eco-friendly production processes. The microrheological properties of silicone rubber play a pivotal role during the foaming process. In this study, Rheolaser Lab (Formulaction, Toulouse, France) was used to conduct in situ examinations for the influence of a crosslinking agent on the microrheological properties of silicone rubber foam for the first time. This study monitors the entire reaction process of silicone rubber foam from liquid to solid, as well as the matching of crosslinking and foaming reactions. Various parameters, including solid–liquid balance, elasticity index, and macroscopic viscosity index, are measured to analyze the microrheological properties of silicone rubber foam. The results show that the silicone rubber foam exhibits good microrheological properties, thereby demonstrating excellent performance at a crosslinking agent content of 2%. Through adjusting the experimental conditions, a sustainable and efficient approach was proposed for better cellular structure control in the industrial preparation of silicone rubber foam.

## 1. Introduction

Silicone rubber foam (SRF) integrates the characteristics of both silicone rubbers and foam materials. SRF exhibits excellent performances, such as being lightweight, having superior temperature resistance, having a low compression set, and having good compression resilience [1,2,3,4]. Therefore, SRF has a multitude of uses in many fields such as the automobile industry, the aerospace field, electronics, and healthcare [5,6]. SRF is usually prepared using high-temperature vulcanization (HTV), which leads to environmental concerns, poor compression set properties, and dimensional instability of the product [7,8]. Thus, the room-temperature-vulcanization (RTV) method is applied to prepare SRF in this study. This method does not require heating during production at room temperature, and the products can be cured and formed under a certain molding pressure.

Room-temperature-vulcanized silicone rubber is a type of SRF that can be based on low-molecular-weight active-straight-chain polysiloxane, in combination with a crosslinking agent, foaming agent, catalyst, and other components [9,10]. The addition–condensation reaction between components produces small molecules of hydrogen gas, which is harmless to the environment [11,12]. The structure and quantity of low-molecular-weight crosslinking agents, as well as the molecular weight of RTV silicone rubber, have a significant impact on the structure of the corresponding RTV SRF [13]. The main function of a crosslinking agent is to generate chemical bonds between linear molecules, such that linear molecules can be connected together to form a network structure, which improves the strength and elasticity of the auxiliary and reduces the possibility of the chain breaking.

Nowadays, most research focuses on the thermal stability and cellular structure of plastic materials. He et al. [14] studied the effects of crosslinking agents on the thermal stability of RTV phenyl silicone rubber and found that it is related to the residual amounts of Si-OH groups. Wang et al. [15] found that water-crosslinked EVM-Si (OC_2_H_5_)_3_ rubber created a secondary network in the system. SiO_2_ nanoparticles could be uniformly dispersed in the matrix. Jiang et al. [16] maintained wet-skid resistance by adding a crosslinking agent to 1,6-bis (N,N′-dibenzyl thiocarbamoyl dithio)-hexane (DBTH) in natural rubber. Li She et al. [13] found that the hydrogen content and chain length of hydrogen-containing silicone oil had an impact on the mechanical properties of silicone rubber.

Microrheology is a new field of rheological methods studying the viscoelastic behavior of soft matter such as emulsions, suspensions, gels, or colloidal dispersions at the micrometer scale [17]. Li et al. [18] obtained the intrinsic crosslinking and gel-breaking rheological kinetics equations, which can effectively describe the crosslinking and gel-breaking processes. Mohsen Ramezani et al. [19] continuously monitored the milk fermentation process, and the test results were confirmed by pH, rheology, and texture monitoring. Moreover, microrheology can be used to detect the impact of antibiotics on gel formation at low concentrations. Tisserand et al. [20] measured the average displacement of the microstructure particles through multispeckle diffusing-wave spectroscopy. They found that this method had the potential to become a method for monitoring physical stability.

At present, there are many studies on the cellular structure, mechanical performance, and rheological properties of SRF. They often use traditional mechanical rheometers to study the rheological properties of SRF, including storage modulus, loss modulus, and complex viscosity [21,22,23]. However, there are few studies on the effect of crosslinking agents on the microrheological properties of SRF. Almost no researchers have applied optical microrheology to SRF prepared via the chemical foaming method yet. In this paper, the microrheological properties of a SRF during the whole preparation process from liquid to solid are studied using Rheolaser Lab. It is also used to analyze their mean square displacement (MSD), solid–liquid balance (SLB), elasticity index (EI), and macroscopic viscosity index (MVI). This provides a certain theoretical basis and technical support for the preparation and use of SRF in the industry.

## 2. Experimental Procedure

### 2.1. Materials

Vinyl-terminated silicone oils with vinyl group (purity ≥ 99%, viscosity = 10 Pa·s) and vinyl-terminated MQ silicone resin (purity ≥ 99%, viscosity = 100 Pa·s) contents of 0.12–0.16% were provided by Jiangxi Bluestar Xinghuo Silicones Co., Ltd. (Jiujiang, China). Hydrogen-containing silicone oil (viscosity = 0.02 Pa·s, purity ≥ 98%, active hydrogen mass fraction = 1.6%) was obtained from Zhejiang Runhe Organic silicone New Material Co., Ltd. (Huzhou, China). Hydroxyl-terminated silicone oil (purity ≥ 98%, hydroxyl value = 5%, viscosity = 0.02 Pa·s,) was provided by Zhonghao Chenguang Research Institute of Chemical Industry Co., Ltd. (Zigong, China). The platinum catalyst (Karstedt’s catalyst, platinum mass fraction = 0.5%) was obtained from Tianjin Umicore Catalyst Co., Ltd. (Tianjin, China). The inhibitor (1-ethynylcycloethanol, purity ≥ 99%) was provided by Guangdong Silicon New Material Co., Ltd. (Guangzhou, China).

### 2.2. Sample Preparation

**Figure 1:**
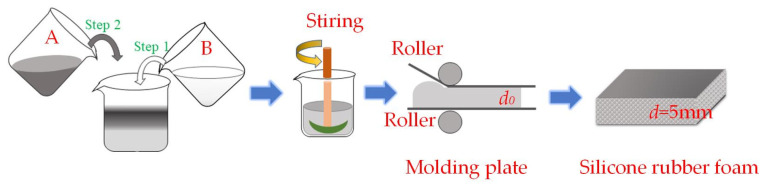
Flow chart for the preparation of SRF.

**Figure 2:**
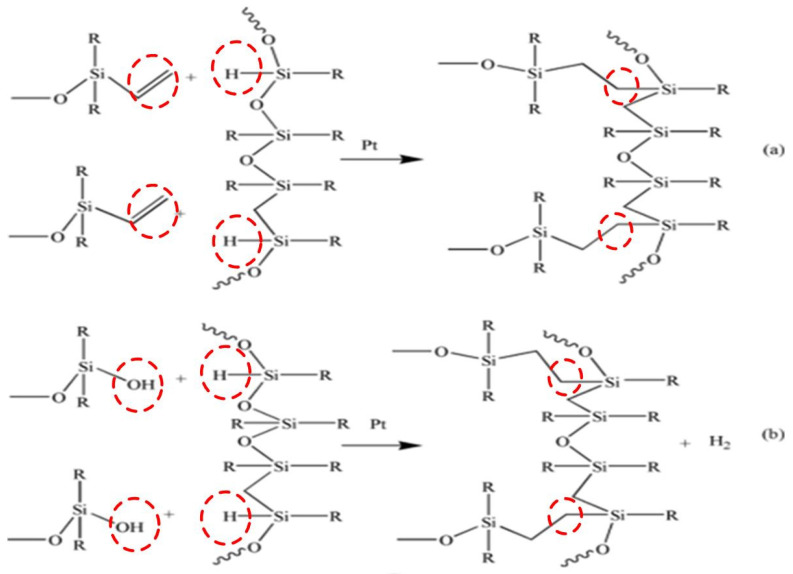
Reaction process of preparing RTV SRF via chemical methods. (**a**) Addition reaction; (**b**) condensation reaction.

According to Table 1, components A and B were stirred with the vacuum defoaming centrifugal mixer at a speed of 800 rpm, respectively. The flow chart for the preparation of SRF is shown in Figure 1. Components A and B were mixed thoroughly at a mass ratio of 1:1. Afterward, the mixture was stirred for 2 min and then left to stand for 30 s. The mixture was quickly poured into the mold at 25 ± 3 °C with a humidity of 40–70%. The thickness of the mixture after passing through the rollers was 1.5 mm. Two minutes later, the molding plate was placed in an oven at 60 °C for 15 min. Then, the SRF samples with a 5 mm thickness were post-cured in the oven at 150 °C for about 15 min. During SRF preparation, hydrogen-containing silicone oil reacted with vinyl-terminated silicone oil and hydroxyl-terminated silicone oil through addition and condensation reactions, respectively (Figure 2).

### 2.3. Characterization

#### 2.3.1. Cellular Structure

We placed the foam samples in liquid nitrogen for approximately 30 min to freeze and fracture, and then obtained the fractured surface sample. The fractured surfaces of the foamed samples were coated with gold. Under an accelerated voltage of 5 kV, the cellular structure was observed through a ZEISS GeminiSEM 500 (ZEISS, Obekochen, Germany) scanning electron microscope (SEM). Cell diameter was measured using Image-Pro Plus 6.0. The sum of all cell diameters was divided by the number of cells (at least 100 cells) in the SEM images to determine the average cell size (*Φ*). The cell density (*N_f_*) of foamed samples was determined as follows [3,24]:

(1) Nf=nM2A32

where *N_f_* is the cell density, *n* is the number of cells, *M* is the magnification, and *A* is the area of microscopic image (cm^2^), which was calculated using Image-Pro Plus 6.0 software.

#### 2.3.2. Crosslinking Density

A VTMR12-010V-I nuclear magnetic resonance crosslinking densimeter designed by Niumai Electronic Technology Co., Ltd. (Niumai, Shanghai, China) was used to measure the crosslinking density of foaming materials according to the industry standard HG/T 2875-1997 “Test Method for Characteristic Values of Crosslinking Density of Rubber-Plastic Microporous Materials” [25]. The effective detection range of the sample was φ8.5 × H20 mm; the magnetic field intensity was 0.5 ± 0.05 T; and the temperature range was controlled from room temperature to 130 °C. The crosslinking density was determined at 30 °C.

#### 2.3.3. Mechanical Properties

**Figure 3:**
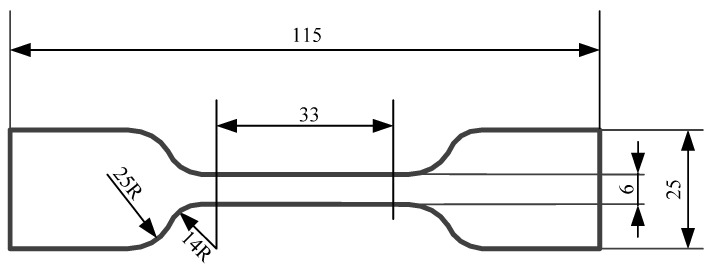
Schematic diagram of the dumbbell test piece.

Mechanical properties of SRF were analyzed through a WDW-10 universal testing machine. A schematic diagram of the dumbbell test piece is shown in Figure 3. The effective part of the sample was 33 mm × 6 mm × 5 mm (length × width × thickness). The tensile rate was 500 mm/min. The specimen should be stretched uniformly during the experiment.

#### 2.3.4. Compression Set

**Figure 4:**
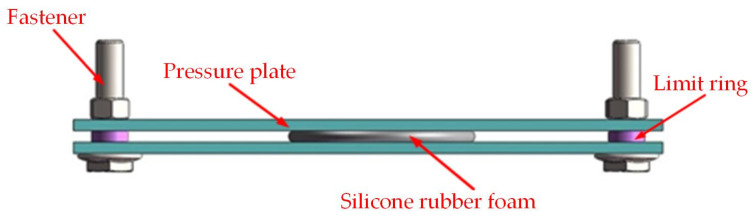
Schematic diagram of customized unit.

The compression set was tested by using a customized unit. The customized unit consisted of three parts: a pressure plate, a fastener, and a limit ring. The parameters were as follows: temperature, 100 °C; compression rate, 50% of the specimen’s thickness; and time, 168 h. A schematic diagram of the customized unit is shown in Figure 4. The specimen size of SRF was φ29 × H5 mm. For each set of data regarding the materials’ properties in our experiment, a minimum of five samples were tested at each crosslinking agent content to obtain average values.

#### 2.3.5. Thermogravimetric Analysis

Thermogravimetric (TG) analysis was performed using a STA449F3 system (NETZSCH, Waldkraiburg, Germany). A 10 ± 0.1 mg sample was placed in an alumina crucible and heated to 800 °C at a heating rate of 20 °C/min in a nitrogen atmosphere. The gas flow rate was set to 100 mL/min to remove corrosive gases involved in the degraded materials.

#### 2.3.6. Rheolaser Lab

A 20 mL mixture comprising components A and B was poured using a plastic pipette along the wall of a flat-bottomed cylindrical glass tube (internal diameter 25 mm, height 70 mm). Rheolaser Lab is an instrument that can observe a wide range of particle sizes, ranging from 0.05 microns to 1 mm for the analyzed samples.

Based on the DWS (diffusing-wave spectroscopy) multispeckle diffusion spectroscopy theory, the Brownian motion velocity of the dispersed phase was converted into an MSD root mean square displacement curve to obtain the microscopic viscoelastic information of the sample, such as the SLB, EI, and MVI parameters. Rheolaser Lab tracked the photon free path I* in real time to study the change procedure of the sample molecular configuration the during the crosslinking or gel-breaking process.

### 2.4. Microrheological Theory

The classical mechanical rheometers may destroy or affect the internal structure of the samples due to dramatic shear. They may exhibit the wall slip phenomenon, which leads to measurement data errors. However, Rheolaser Lab can measure the viscoelastic behavior of samples in fully static conditions using multispeckle diffusion spectroscopy technology. It has no mechanical shear stress during the measurement process and can measure particularly fragile samples [26]. It advantages consist of providing convenient, accurate, and reliable results, and being non-contact in studying soft materials’ rheology.

**Figure 5:**
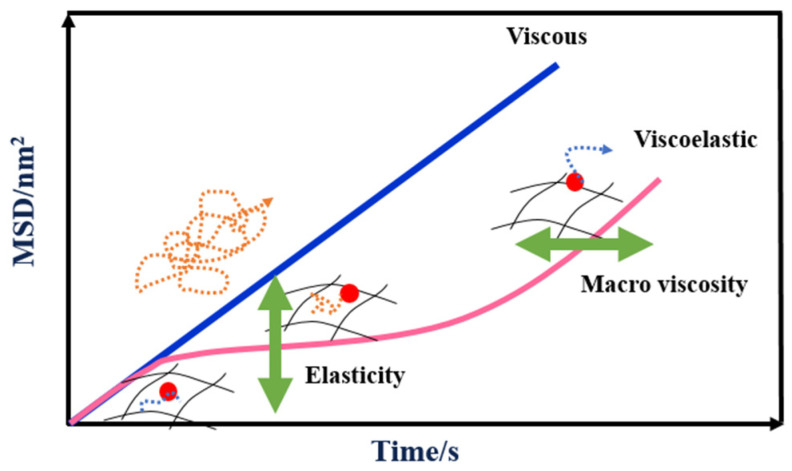
MSD curves of purely viscous and viscoelastic samples.

The MSD curve can characterize the Brownian motion of tracer particles. Tracer particles are fumed silica with an average particle size of 1 μm and a content of about 13%. Figure 5 shows MSD curves of different samples at the decorrelation time. The decorrelation time is the measurement time, which is used to follow the change in the speckle image [27]. It can be seen that in the case of a fully viscous sample, the MSD increases linearly with decorrelation time because the particles are completely free to move in the sample. However, the MSD curve of a viscoelastic sample can be divided into three stages based on the decorrelation time. Initially, the MSD curve shows a linear development. The slope of the MSD curve decreases and eventually reaches a plateau, which is a characteristic of the sample’s elasticity. As the decorrelation time increases, the particles are able to escape from the “cage” and the MSD starts to grow linearly again. This linear growth corresponds to the macroscopic viscosity and represents the speed at which particles move within the sample. Parameters like SLB, EI, and MVI extracted from the MSD curves can be used to characterize the viscoelastic properties of samples.

SLB corresponds to the MSD slope at a short decorrelation time. The physical meaning of SLB is the same as the tanδ obtained by a rotational rheometer [28].

EI is calculated based on the elastic plateau value, which is the reciprocal of the height of the MSD plateau. There is a positive correlation between *EI* and elastic modulus *G*′*_plateau_* at the platform. *EI* and elasticity have the same change trend. The *EI* of SRF is determined according to the following equations [29]:

(2) $${G^{\prime }}_{plateau}=\frac{K_{b}T}{h\pi a}$$

(3) $$EI=\frac{1}{h\pi a}={G^{\prime }}_{plateau}\;constant$$

where *G*′*_plateau_* is the elastic modulus at the platform (Pa); *h* is the height value at the plateau of MSD; *K_b_* is the Boltzmann constant (1.380649 × 10^−23^ JK^−1^); *a* is the particle mean diameter (μm); and *EI* is the elasticity index (nm^−2^).

MVI is the viscosity index at zero shear rate, which is the reciprocal of the MSD curve’s slope with a long decorrelation time. This slope is related to the macroscopic viscosity of the samples. The MVI of SRF is determined according to the following equations [28]:

(4) $$\eta_{macroscopic}=\frac{K_{b}T}{6D_{m}\pi a}$$

(5) $$MVI=\frac{1}{6D_{m}a}=\eta_{macroscopic}constant$$

where 1/6*D_m_* is the inverse of the slope of MSD curves; *η_macroscopic_* is the macroscopic viscosity (Pa·s); *K_b_* is the Boltzmann constant (1.380649 × 10^−23^ JK^−1^); *a* is the particle mean diameter (μm); and *D_m_* is the diffusion coefficient (nm^2^·s^−1^).

## 3. Results and Discussion

### 3.1. Effect of Crosslinking Agent on Cellular Structure of SRF

In the formula component of SRF, hydrogen-containing silicone oil is selected as the crosslinking agent. During the preparation of SRF, the function of Si-H in the crosslinking agents is to participate in an addition reaction with the CH_2_=CH- bond in the vinyl silicone oil, forming Si–C bonds (as shown in Figure 2a). At the same time, Si–H in the crosslinking agent is condensed with Si-OH in hydroxyl silicone oil to form Si–O–Si bonds (as shown in Figure 2b) and produces small molecules of hydrogen gas, ultimately forming the cellular structure of SRF.

**Figure 6:**
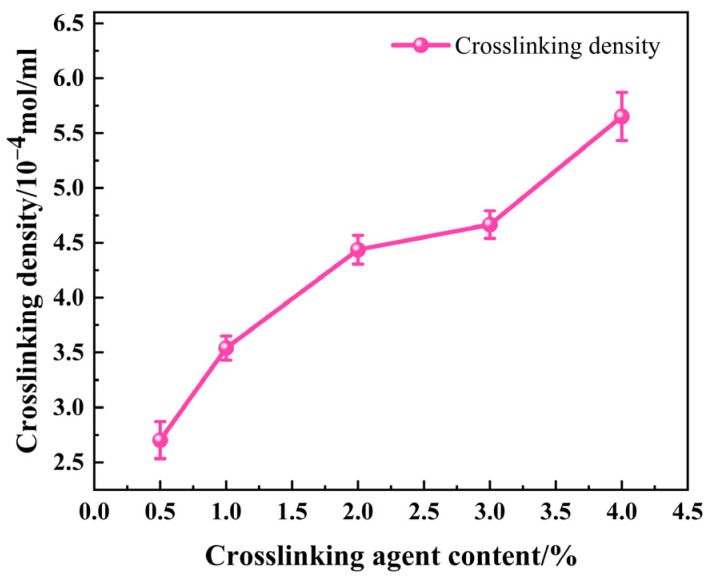
Effect of the crosslinking agent content on the crosslinking density of SRF.

Figure 6 shows the effect of the crosslinking agent content on the crosslinking density of SRF. It can be seen that when the content of a crosslinking agent is 0.5%, 1%, 2%, 3%, and 4%, the crosslinking density of SRF is 2.765 × 10^−4^ mol/mL, 3.423 × 10^−4^ mol/mL, 4.451 × 10^−4^ mol/mL, 4.667 × 10^−4^ mol/mL, and 5.602 × 10^−4^ mol/mL, respectively. The crosslinking density of SRF increases with the increase in crosslinking agent content, and the growth is 23.8%, 30.0%, 4.85%, and 20.0%, respectively. This is because as the crosslinking agent content increasing, the content of Si–H increases and the crosslink bond quantity in SRF increases. Therefore, more crosslinked network structures are formed, and the crosslinking density is higher.

**Figure 7:**
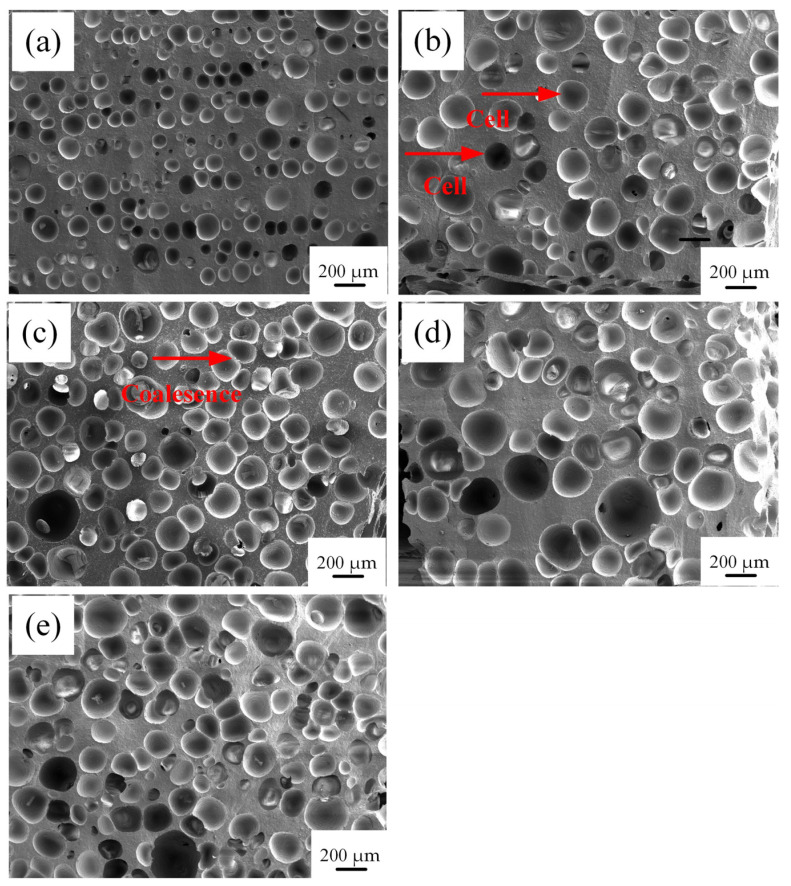
Effect of the crosslinking agent content on the cellular structure of SRF. (**a**) 0.5%; (**b**) 1%; (**c**) 2%; (**d**) 3%; (**e**) 4%.

**Figure 8:**
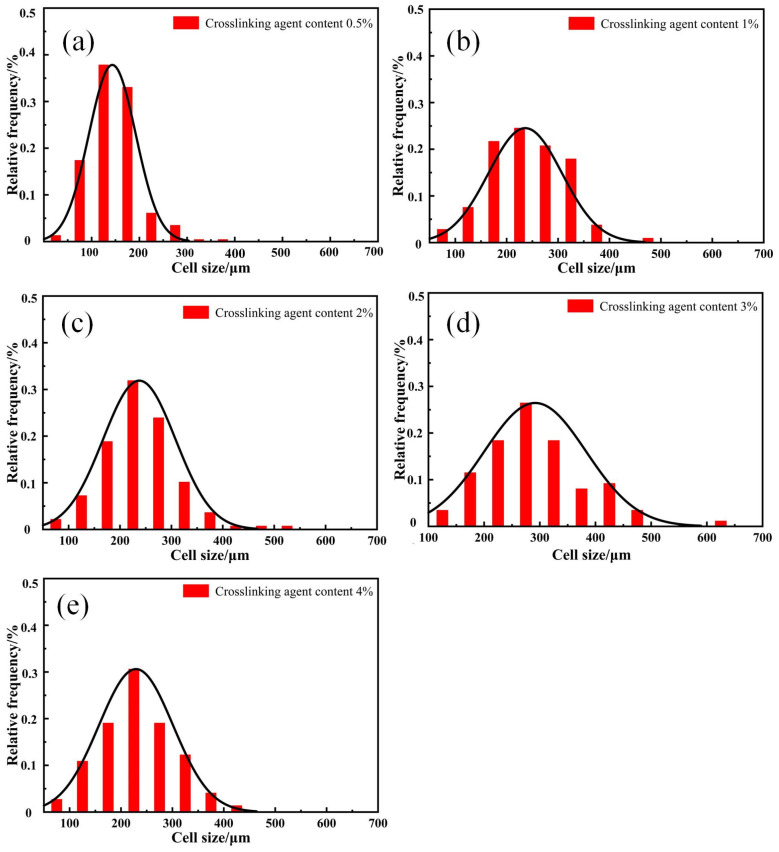
Effect of crosslinking agent content on the cell size distribution of SRF. (**a**) 0.5%; (**b**) 1%; (**c**) 2%; (**d**) 3%; (**e**) 4%.

Figure 7 and Figure 8 show the effect of crosslinking agent content on the cellular structure and cell size distribution of SRF. The foam cell shape is mostly circular or elliptical, as is shown Figure 7.

With the increase in crosslinking agent content, the number of cells in SRF gradually increases, and the overall size of the cells shows an increasing trend. The thickness of the cell wall between the cells gradually decreases. When the crosslinking agent content increases from 0.5% to 4%, the average pore size of SRF increases from 143 μm to 229 μm. When the crosslinking agent content is 3%, the cell size of SRF is the largest, with a size of approximately 291 μm. At the same time, it is found that when the content of the crosslinking agent is low, there are more intact cell walls and smaller cell diameters; with the further increase in crosslinking agent content, the cell walls of SRF start to become incomplete, and there is much cell coalescence, resulting in a decrease in the skeleton thickness between pores. As observed in Figure 8, with the increase in crosslinking agent content, the cell size distribution of SRF widens.

In order to quantitatively study the influence of the crosslinking agent content on the cellular structure of SRF, the foam density is measured by computer software, and the cellular structure parameters are analyzed, as shown in Table 2. With the increase in crosslinking agent content, although the cell size is larger, the porosity has an increasing trend, from 47% to 70%. At the same time, we can obviously observe the phenomenon of cell coalescence. As the cell size increases, the cell wall becomes thinner. The peak of the cell size distribution curve moves to a larger-sized region, and the cell size distribution gradually widens with increasing crosslinking agent content. The widening of the distribution is mainly due to the increase in the amount of hydrogen-containing silicone oil, the increase in the number of bubbles, and the coalescence phenomenon, resulting in an increase in cell heterogeneity [30]. We find that hydrogen gas is released due to the condensation reaction between components during the preparation of SRF. From the SEM cell structure observation, it can be seen that very few bubbles grow on their own. The phenomenon of controlling cell growth is cell coalescence. Jawhar [31] prepared silicone foam using a two-component system. Their mechanism was consistent with ours.

### 3.2. Effects of a Crosslinking Agent on the Mechanical Properties of SRF

**Figure 9:**
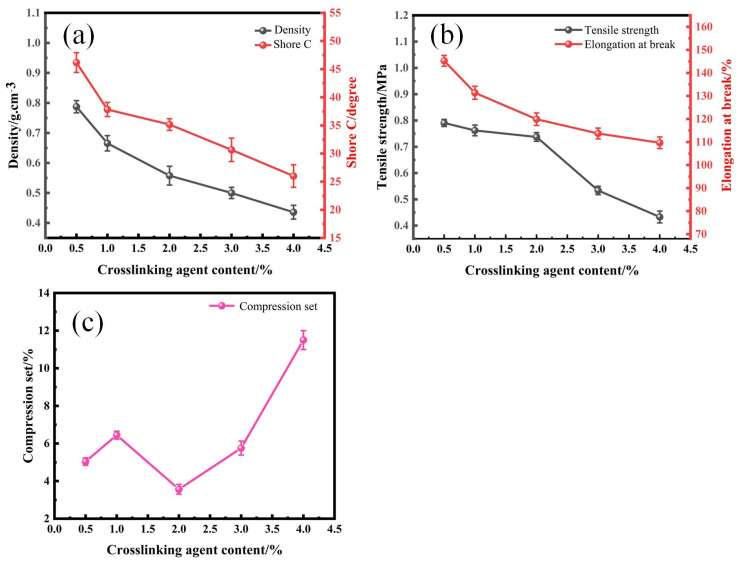
Effects of a crosslinking agent on the mechanical properties of SRF. (**a**) Density and shore C; (**b**) tensile strength and elongation at break; (**c**) compression set.

The selected crosslinking agent of SRF is hydrogen-containing silicone oil with a hydrogen content of 1.6%. The effects of crosslinking agent content on the mechanical properties of SRF are shown in Figure 9. With the increase in crosslinking agent content, the density, shore C, tensile strength, and elongation at break of SRF decreased. With the increase in crosslinking agent content from 0.5% to 4%, the density decreased from 0.765 g/cm^3^ to 0.437 g/cm^3^. Shore C decreased from 44.5 degrees to 24 degrees. Tensile strength decreased from 0.79 MPa to 0.44 MPa. The elongation at break of SRF decreased from 145.8% to 110.9%. With the increase in hydrogen-containing silicone oil in the crosslinking agent during the reaction process, more H_2_ content was generated in the system. This causes an increase in cell size, resulting in more pores and thinner cell walls, leading to coalescence between cells. This ultimately leads to a decrease in the density, hardness, and tensile strength of SRF.

The compression set represents the resistance to compression deformation of materials such as foam and rubber. The smaller the compression set value, the stronger the material’s resistance to compression deformation will be. The higher the compression set value, the weaker the compressive resistance of the material will be. Figure 9c shows the effect of the crosslinking agent content on the compression set of SRF. The compression set of SRF generally shows an increase with the increase in crosslinking agent content, except for the abnormal reduction that occurs when the concentration increases from 1.0% to 2.0%. When the crosslinking agent content is 0.5%, 1%, 2%, 3%, and 4%, the compression set of SRF is 4.82%, 6.53%, 3.29%, 5.36%, and 11%, respectively.

The reason for this is that as the crosslinking agent content increases, the crosslinking bonds of the prepared SRF increase in number, the crosslinking density increases, the number of cells formed is larger, and the cell size is relatively uniform. Moreover, the compression set is related to the viscoelasticity of the material and its ability to restore itself to its original state. When the crosslinking agent content is 0.5% and 1%, SRF has a lower crosslinking density and uneven cell walls. Its ability to recover to its original state is average during the compression deformation test. At a crosslinking agent content of 2%, the crosslinking density of SRF increases, the cell wall thickness is moderate, the cell size is more uniform, and there is less cell coalescence. The compression resistance is good. At a crosslinking agent content of 3% and 4%, the crosslinking density further increases, and all the cells are enclosed inside silicone rubber. The cell size shows an increasing trend, with a thinner wall thickness. The phenomenon of cell coalescence increases, causing the SRF to be prone to collapse and making it unable to restore itself to its original state during the compression deformation test. This leads to a greater compression set.

Therefore, when the crosslinking agent content is 2%, the arrangement of SRF cells is relatively tight, and the thickness of the formed cell wall is also relatively moderate. At the same time, the crosslinking reaction and foaming reaction during the preparation of SRF reach equilibrium. The crosslinking reaction is accompanied by a foaming reaction during the preparation of SRF. If the crosslinking rate is too low or the foaming rate is too high, the incomplete crosslinking of silicone rubber cannot effectively enclose the gas. This leads to the hydrogen gas emission and cell coalescence, forming big cells or open cells. In the other case, if the crosslinking rate is too high or the foaming rate is too low, the rate of cell growth is low, and the polymer melt has a greater constraint on the bubbles, leading to the formation of small cells or closed cells [32]. An excellent performance of SRF can be obtained only when the crosslinking reaction and the foaming reaction are well matched. Therefore, new siloxane or carbosilane linkages form a polymer network in the crosslinking SRF [33]. The elastic modulus of the system may be the highest and the ability of SRF to recover to its original state is the strongest. It has the strongest ability to resist permanent deformation caused by compression. The compression set of SRF is the lowest.

### 3.3. Effect of a Crosslinking Agent on the Thermal Stability of SRF

**Figure 10:**
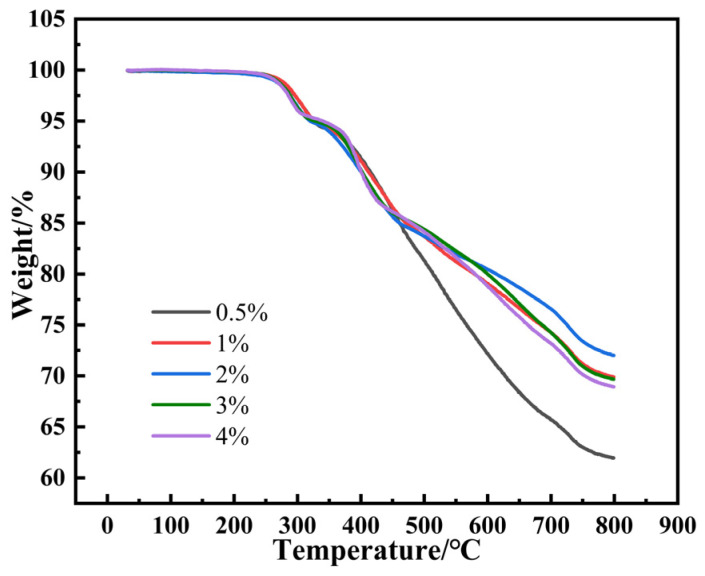
TG curves of SRF with various crosslinking agent contents.

Figure 10 shows the TG curves of SRF with different contents of the crosslinking agent. It can be seen that the weight loss of SRF is divided into three stages. The first stage (240–330 °C) is the breaking of the main chain, which forms the cyclic siloxane in the process [34,35]. The second stage (330–475 °C) is the decomposition of side chains [36]. The third stage (475–800 °C) is the carbonization of SRF [37]. The thermal decomposition temperature of SRF shows a gradually decreasing trend with the increase in crosslinking agent content. The temperature at a thermal weight loss of 10 wt.% (T10) decreases from 415 °C to 400 °C. With the increase in crosslinking agent content, the temperature at a thermal weight loss of 20 wt.% (T20) first increases and then decreases. When the crosslinking agent content is at 2%, the temperature at a thermal weight loss of 20 wt.% (T20) is the highest, approximately 600 °C. It can be seen from Figure 10 that the residues of SRF at 800 °C is 61.9%, 69.9%, 72.0%, 69.7%, and 68.9% for crosslinking agent contents of 0.5%, 1%, 2%, 3%, and 4%, respectively. Finally, it is concluded that the stability of SRF is best when the crosslinking agent content is 2%.

### 3.4. Effect of a Crosslinking Agent on the Microrheological Properties of SRF

**Figure 11:**
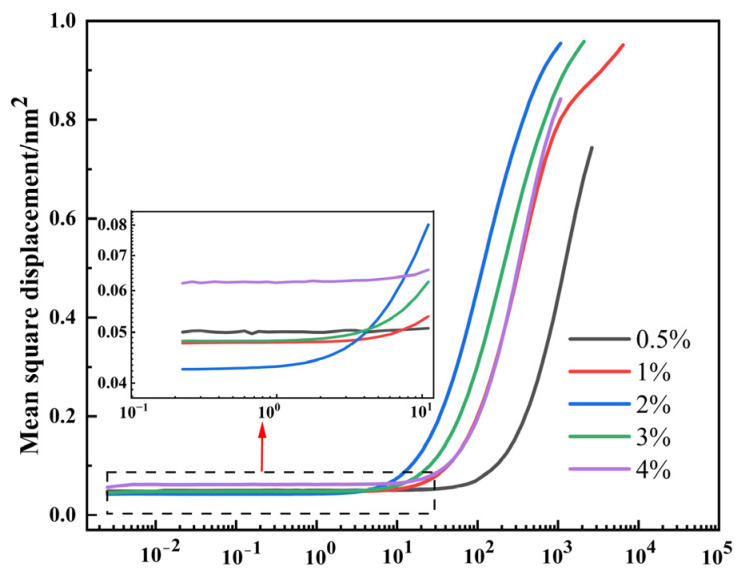
MSD curves of SRF with various crosslinking agent contents.

Figure 11 displays the MSD curves of SRF with various crosslinking agent contents. The MSD curves are consistent with the trend of a viscoelastic sample of Figure 5. As shown in Figure 11, MSD curves are categorized into three stages based on the decorrelation time, which comprise the nearly linear region, the plateau region, and the later linear region, respectively. With the increase in crosslinking agent content, the value of the plateau shows a trend of first decreasing and then increasing. When the crosslinking agent content is 0.5%, 1%, 2%, 3%, and 4%, the value of the plateau is 0.050 nm^−2^, 0.048 nm^−2^, 0.043 nm^−2^, 0.048 nm^−2^, and 0.062 nm^−2^, respectively. The lower the value of the plateau, the better the viscoelasticity of SRF. When the crosslinking agent content is 2%, the value of the plateau is the lowest. The internal structure of SRF is the most dense. From the graph, it can also be seen that with the increase in crosslinking agent content, the slope of the MSD curve’s later linear region shows a trend of first increasing and then decreasing. The shallower the slope, the higher the viscosity of the system, and the slower the particle motion speed. As shown in Figure 11, with the increase in crosslinking agent content, the time required for a particle to reach the same movement distance shows a trend of first decreasing and then increasing. When the crosslinking agent content is 2%, the system viscosity is the lowest and the particle movement velocity is the fastest.

The SLB value represents the solid–liquid balance state of the sample, which can indicate whether the sample has more solid or liquid characteristics. When SLB = 0, the system is a pure elastic solid. The system with 0 < SLB < 0.5 exhibits partial elastic solid properties (gel behavior). The critical point of liquid–solid conversion is SLB = 0.5. A system with 0.5 < SLB < 1 exhibits partial viscous liquid characteristics. The system with SLB > 1 exhibits fully viscous fluid characteristics. The smaller the SLB value, the lower the surface particle motion rate, and the more solid characteristics the sample exhibits [38].

**Figure 12:**
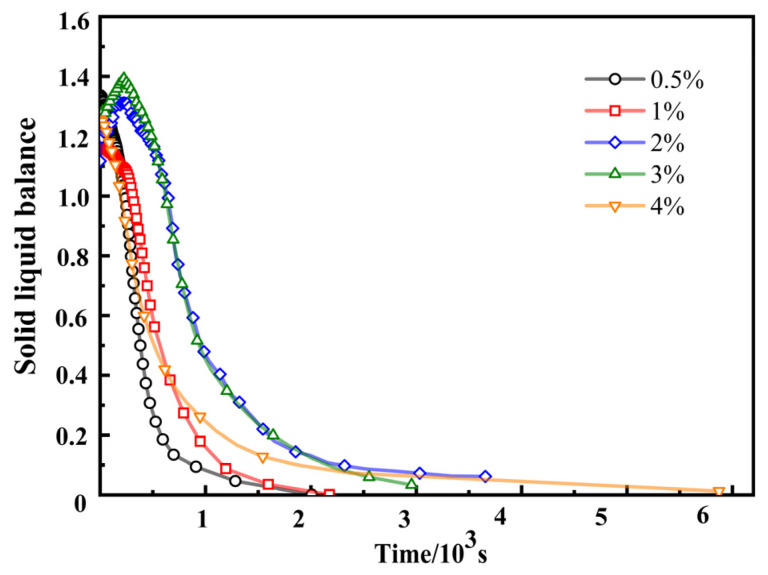
Effect of crosslinking agent content on the SLB of SRF.

The effect of the crosslinking agent content on the SLB of SRF is shown in Figure 12. It can be seen from Figure 12 that SLB curves first increase and reach a maximum, then decrease, and finally stabilize as the time step increases. This is because during the early crosslinking process, viscosity increases faster than elasticity, which is called “weak crosslinking”. In the later stage of crosslinking, elasticity increases faster than viscosity, which is called “strong crosslinking” [39]. It is fully verified that during the preparation of SRF, the polymer melt is initially liquid and, as time passes, it eventually becomes solid. With the increase in crosslinking agent content, the time required for the SLB value to reach the liquid–solid conversion critical point (SLB = 0.5) is also first extended and then shortened. When the crosslinking agent content is 2%, the time required for the SLB value of SRF to reach 0.5 is the longest, approximately 984 s. As the content of crosslinking agent increases, the time required for the entire SLB curves to transition from solid to liquid also increases. SLB curves monitor the characteristics of SRF throughout the entire reaction process from liquid to solid. This can help us to understand more about the preparation of SRF.

**Figure 13:**
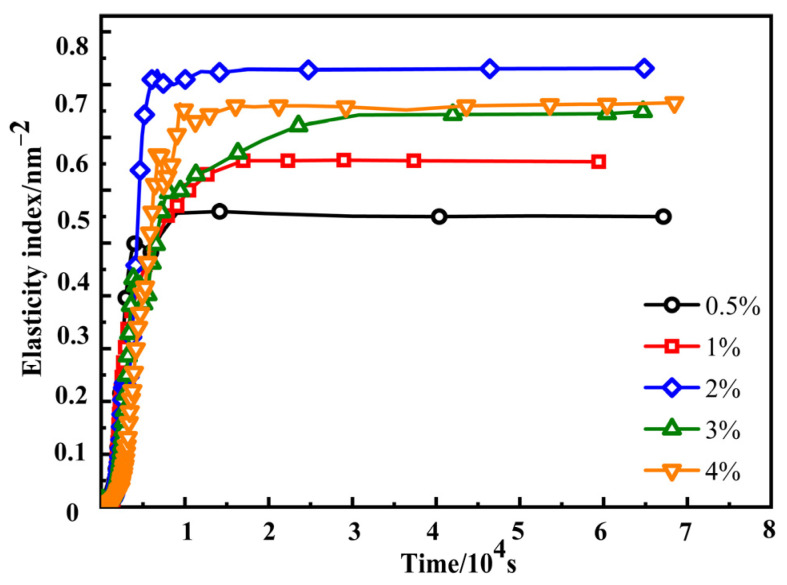
Effect of crosslinking agent content on the EI of SRF.

The EI is used to describe the elastic properties of the sample. The storage or elastic modulus are equivalent terms for G′ [40]. The formula for the EI shows that there is a positive correlation between EI and the elastic modulus G′_plateau_ at the platform. The higher the EI value, the higher elasticity of the sample, and the stronger the material’s resistance to compression deformation. The effect of crosslinking agent content on the elasticity index (EI) of SRF is shown in Figure 13 and Table 3. It can be seen from Figure 13 that as the time step increases, EI curves first increase and then stabilize. This is because Si-H in the crosslinking agent undergoes an addition–condensation reaction with Si-CH=CH_2_ in the vinyl silicone oil and Si-OH in the hydroxyl silicone oil to form new chemical bonds. The “cage” or “network structure” in the system gradually forms and the number of “cages” increases [21]. As a result, the “cage” space gradually becomes smaller, and particle motion is constrained, resulting in an increase in the elasticity of the system [41].

When the crosslinking agent content is 0.5% and 1%, the EI’s stability value is relatively small, indicating that the elasticity of SRF is the worst. When the content of the crosslinking agent is 2%, the relative stability value of EI is the highest, indicating that the elasticity of SRF is the best. Compared to the case of SRF at a crosslinking agent content of 2%, the EI of SRF at a crosslinking agent content of 3% and 4% is lower. SRF does not necessarily have better elasticity with higher crosslinking agent content during the preparation process. Therefore, we need to choose a suitable content to meet the requirements.

**Figure 14:**
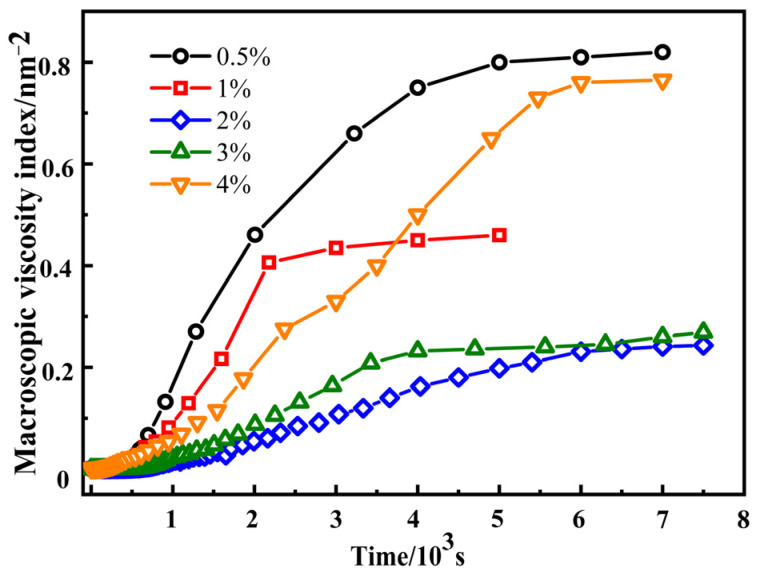
Effect of crosslinking agent content on the MVI of SRF.

MVI is a quantitative description of the macroscopic viscosity of a sample [25,42]. The higher the MVI value, the higher the viscosity of the sample, and the lower the motion speed of the particle. Therefore, the particle needs a longer time to reach the sample to move a given distance. The effect of the crosslinking agent content on the MVI of SRF is shown in Figure 14 and Table 4. It can be seen from Figure 14 that as the time step increases, the MVI curves of SRF first increase and then stabilize. This indicates that the viscosity of SRF shows an increasing trend. With the increase in crosslinking agent content, the stable MVI value of SRF first decreases and then increases. When the crosslinking agent content is 0.5%, 1%, 2%, 3%, and 4%, the MVI stability value is 0.82 nm^−2^, 0.45 nm^−2^, 0.24 nm^−2^, 0.27 nm^−2^, and 0.77 nm^−2^, respectively. The lower the MVI value, the lower viscosity of the sample. The particles encounter lower resistance during the crosslinking and foaming reaction. The time required for the sample to travel a given distance is shorter. The stable MVI value of SRF is the smallest at a crosslinking agent content of 2%. This indicates that the system’s viscosity is the lowest and particle motion speed is the highest.

Through the analysis of the EI and MVI results of SRF, it was found that when the crosslinking agent content was 0.5% and 1%, the EI value was small, while the MVI value was large. During the foaming process, the cells are subjected to greater resistance, and SRF has lower viscoelasticity, which is not conducive to the growth and nucleation of cells. Therefore, the cell size is smaller, and the cell wall thickness is thicker. When the crosslinking agent content is 2%, the EI value is the highest, the MVI value is the lowest, the elastic modulus is the highest, and the viscosity is the lowest. During the foaming process, the cells are subjected to a lower level of resistance, and the viscoelasticity is the best. Therefore, the cell size is uniform, the cell wall thickness is relatively consistent, and there is less cell coalescence. Compared to a crosslinking agent content of 2%, at a crosslinking agent content of 3% and 4%, the EI value decreases and the MVI value increases, resulting in a decrease in the elastic modulus and an increase in viscosity. This leads to lower viscoelasticity and greater resistance, enclosing all cells inside silicone rubber, thus exacerbating the phenomenon of cell coalescence [43,44].

Therefore, the viscoelasticity of SRF is excellent at a crosslinking agent content of 2%. The higher viscoelasticity can reach equilibrium and maintain a good balance between crosslinking and foaming reactions. This means that the cellular structure tends to be spherical and the size is more uniform, which promotes the stability of the cellular structure and inhibits the deterioration of the cellular structure [23]. We find that the viscoelastic properties of silicone rubber play a pivotal role in the foaming process, which is closely associated with the crosslinking structure of silicone rubber. A similar opinion was found in reference [23].

In general, SRF not only demands good viscoelasticity but also requires a good balance between crosslinking and foaming reactions during the entire reaction process from liquid to solid. An excellent performance of SRF can thus be obtained.

## 4. Conclusions

In this study, the effect of a crosslinking agent on the microrheological properties of SRF prepared via a green process was discussed through in situ examinations. We clarified the cellular structure, mechanical properties, and microrheological properties of SRF using SEM, a universal testing machine, a nuclear magnetic resonance crosslinking densimeter, and Rheolaser Lab, respectively. The following conclusions were drawn:

(1) During the preparation of SRF, few cells grow on their own due to the hydrogen gas generated via the condensation reaction between components, resulting in the formation of cells. The main phenomenon that controls cell growth is cell coalescence.
(2) With the increase in crosslinking agent content, the number of cells in SRF gradually increases, and coalescence occurs between cells. The porosity has an increasing trend. The crosslinking density of SRF increases, while the hardness, tensile strength, and elongation at break show decreasing trends.
(3) By studying the microrheological properties of SRF, it is found that the viscoelasticity of SRF is best at a crosslinking agent content of 2%, the crosslinking and foaming reactions reach equilibrium, and they maintain a good balance. Therefore, its comprehensive performance is the best.
(4) The entire reaction process and microrheological properties of SRF from the liquid to solid can be detected using Rheolaser Lab. To a certain extent, this can provide important data and support for the industrial production of SRF.

## Figures and Tables

**Table 1:** Experimental formulation of SRF.

Component	Materials	Viscosity/Pa·s	Mass Fraction/%
A	Vinyl-terminated silicone oil	10	25
	Vinyl-terminated MQ silicone resin	100	24
	Hydroxyl-terminated silicone oil	0.02	0.5
	Catalyst	−1	0.5
B	Vinyl-terminated silicone oil	10	25
	Vinyl-terminated MQ silicone resin	100	23.95
	Hydrogen-containing silicone oil	0.02	0.5, 1, 2, 3, 4
	Inhibitor	0.1	0.05

**Table 2:** Effect of crosslinking agent content on the cellular structure of SRF.

Cellular Structures	a	b	c	d	e
Cell average size (μm)	143	236	237	291	229
Cell density (cells/cm^3^)	5.41 × 10^9^	1.69 × 10^9^	2.29 × 10^9^	1.26 × 10^9^	3.03 × 10^9^
Foaming density (g/cm^3^)	0.765	0.637	0.567	0.494	0.437
Porosity (%)	47	56	61	66	70

**Table 3:** EI value of SRF with various contents of the crosslinking agent.

Crosslinking Agent (%)	0.5	1	2	3	4
EI (nm^−2^)	0.56	0.66	0.83	0.74	0.77

**Table 4:** MVI value of SRF with various contents of the crosslinking agent.

Crosslinking Agent (%)	0.5	1	2	3	4
MVI (nm^−2^)	0.82	0.45	0.24	0.27	0.77

## Data Availability

Data are contained within the article.
